# Lobe‐specific analysis of perioperative chemotherapy for non‐small cell lung cancer patients

**DOI:** 10.1002/cam4.6319

**Published:** 2023-07-05

**Authors:** Xi Lei, Tong Li, Fuling Mao, Fan Ren, Quanying Tang, Weibo Cao, Lingling Zu, Song Xu

**Affiliations:** ^1^ Department of Lung Cancer Surgery Tianjin Medical University General Hospital Tianjin China; ^2^ Tianjin Key Laboratory of Lung Cancer Metastasis and Tumor Microenvironment, Lung Cancer Institute, Tianjin Medical University General Hospital Tianjin China

**Keywords:** adjuvant chemotherapy, lobe‐specific, neoadjuvant chemotherapy, NSCLC, SEER database

## Abstract

**Objectives:**

Perioperative cisplatin‐based chemotherapy decreases the risk of death over surgery alone and is a standard of care. Here, we examined perioperative chemotherapy indications for stage IB‐III non‐small cell lung cancer (NSCLC) patients according to lobe‐specific analysis.

**Methods:**

Resectable NSCLC patients with stage IB–III who received perioperative chemotherapy with and without radiotherapy after lung resection were identified from the SEER database. Propensity score matching (PSM) analysis was performed to reduce the inherent bias of retrospective studies. The Kaplan–Meier method and log‐rank tests were used to assess the differences in overall survival (OS).

**Results:**

The study enrolled 23,844 patients before PSM. The perioperative chemotherapy group had better OS than the nonperioperative chemotherapy group in stage IB–III NSCLC patients before and after PSM. However, subgroup analysis according to stage demonstrated that perioperative chemotherapy did not markedly benefit patients with stage IB. Furthermore, lobar subgroup analysis did not show survival advantages in primary tumors located in either the right middle lobe in stages II and III NSCLC or the right lower lobe in stage III NSCLC.

**Conclusions:**

Lobe‐specific perioperative chemotherapy is recommended in NSCLC patients. For stage IB NSCLC, right middle lobe NSCLC from stage IB‐III and right lower lobe NSCLC from stage III, perioperative chemotherapy might not confer survival benefits.

## 1 INTRODUCTION

In patients with early‐ and mid‐stage NSCLC, complete surgical resection is the most effective treatment.^1^ However, 30%–70% of patients relapse after resection.^2^, ^3^ Therefore, perioperative chemotherapy may improve the surgical outcome and thus patient survival.^4^, ^5^, ^6^ Several randomized control studies (RCT) and a pooled analysis of trials indicate that cisplatin‐based adjuvant chemotherapy improves the 5‐year survival by 5%,^7^, ^8^, ^9^ although the results are controversial for patients with stage IB, as adjuvant chemotherapy, one of the perioperative chemotherapy options, is recommended as a standard treatment for early‐stage NSCLC patients by international guidelines.^10^, ^11^ Neoadjuvant chemotherapy, the other part of perioperative treatment, was not evaluated as extensively but still demonstrated an absolute benefit of 5% at 5‐years, which was equal to that of adjuvant chemotherapy.^12^, ^13^ Overall, studies addressing the role of perioperative chemotherapy showed that it confers a survival benefit.

Differences in the efficacy of pemetrexed between squamous cell carcinoma and nonsquamous cell carcinoma led to the assessment of precision chemotherapy to improve the efficacy of treatment. Several factors, such as stage, chemotherapy regimens, and histologic type, need to be considered in the selection of perioperative chemotherapy to maximize the survival benefits of NSCLC patients. However, tumor location, an important factor affecting the prognosis of NSCLC, is not included in decisions regarding chemotherapy. Because tumors in different lobes are associated with different prognoses after surgery, radiotherapy, and immunotherapy,^14^, ^15^, ^16^, ^17^ we examined the survival benefits of perioperative chemotherapy according to the lobes affected to provide precise perioperative chemotherapy options that increase the survival benefit to patients.

## 2 PATIENTS AND METHODS

### 2.1 Ethics statement

SEER data are available to eligible individuals from the National Cancer Institute (NCI) upon completion of a data use agreement. We completed a data use agreement to access the data reported in this manuscript. A data use agreement per NCI/SEER policies was signed in order to obtain approval to access deidentified SEER public‐use data through SEER*Stat.

### 2.2 Patient selection

Cases were extracted from the SEER database (SEER stat 8.2.9.2). The inclusion criteria were as follows^1^: patients diagnosed with NSCLC stage IB–III^2^; patients who underwent lobectomy or sublobar resection^3^; patients who received chemotherapy but no radiotherapy^4^; location of the primary tumor was exactly in the right upper lobe, right middle lobe, right lower lobe, left upper lobe, or left lower lobe. The exclusion criteria were as follows^1^: patients who underwent surgery other than lobectomy or sublobar resection^2^; patients with missing information regarding survival time and stage^3^; patients whose stage information was not detailed. The extracted information included age, race, sex, location of the primary tumor, histologic type, stage, surgery type, number of resected lymph nodes (LNs), chemotherapy record, vital status records, and survival months.

### 2.3 Statistical analysis

Pearson's chi‐square tests were used to compare categorical variables. Overall survival (OS) was assessed using the Kaplan–Meier method and compared using the log‐rank test. The correlations between clinicopathological characteristics and OS were estimated by Cox proportional hazards regression models, and the hazard ratio (HR) and corresponding 95% confidence interval (CI) were calculated. To balance multiple characteristics in retrospective studies to approximate a random experiment, PSM was performed between the nonperioperative chemotherapy group and perioperative chemotherapy group in stage IB‐III patients. Statistical analyses and PSM were performed using SPSS (IBM SPSS Statistics version 26.0, Chicago, IL, US). Statistical significance was considered at a two‐sided *p* < 0.05.

## 3 RESULTS

### 3.1 Patient characteristics

The patient characteristics are described in Table 1. A flowchart of the selection process is shown in Figure S1. As shown in Table 1, the median age was 70 years, and perioperative chemotherapy was performed more often in younger people (*p* < 0.001). There was no significant difference in sex between the two arms (*p* = 0.357) and white people accounted for 84% of the population. Lung adenocarcinoma (LUAD) was the predominant histology, accounting for 40.52% of the cohort. There were significant differences in the prevalence of LN metastases between patients who received perioperative chemotherapy and those treated with surgery alone (*p* < 0.001). Consistent with previous studies, the right upper lobe (RUL) was the most common tumor site, accounting for 32.16%, and the remaining tumors were located in the left upper lobe (LUL), right lower lobe (RLL), left lower lobe (LLL), and right middle lobe (RML). Resection consisted of lobectomy in 84.45% and sublobar resection in 15.55% of patients; resection combined with LN dissection (88.69%) was the main treatment.

**TABLE 1:** Baseline characteristics of NSCLC patients with stage IB‐III.

	Estimate	Before PSM			Estimate	After PSM		
		No	Yes	*p* value		No	Yes	*p* value
	23,844	16,857	6987		9850	4925	4925	
Age								
≤70	12,912	8147	4765	<0.001	6190	3101	3089	0.802
>70	10,932	8710	2222		3660	1824	1836	
Sex								
Male	11,629	8189	3440	0.357	4977	2484	2493	0.856
Female	12,215	8668	3547		4873	2441	2432	
Race								
White	20,036	14,223	5813	0.078	8588	4294	4294	0.994
Black	2115	1463	652		720	359	361	
Other	1693	1171	522		542	272	270	
Histologic type								
LUSC	5942	4336	1606	<0.001	2319	1160	1159	0.992
LUAD	9661	6547	3114		4019	2012	2007	
OC	8241	5974	2267		3512	1753	1759	
Location								
Right upper lobe	7668	5409	2259	0.011	3285	1647	1638	0.999
Right middle lobe	1409	1052	357		431	215	216	
Right lower lobe	4702	3307	1395		1952	977	975	
Left upper lobe	6148	4358	1790		2524	1257	1267	
Left lower lobe	3917	2731	1186		1658	829	829	
No. of resected lymph nodes								
0	1630	1336	294	<0.001	413	205	208	0.989
>1–3	3057	2405	652		845	427	418	
≥4	18,091	12,365	5726		8320	4157	4163	
Other	1066	751	315		272	136	136	
Surgery Type								
Sub	3708	2977	731	<0.001	900	452	448	0.889
Lobectomy	20,136	13,880	6256		8950	4473	4477	
Stage								
IB	11,174	9817	1357	<0.001	2654	1328	1326	0.993
IIA	4817	2535	2282		2995	1491	1504	
IIB	3952	2653	1299		2049	1029	1020	
IIIA	3710	1780	1930		2093	1046	1047	
IIIB	191	72	119		59	31	28	
LN Metastases								
No	17,013	13,866	3147	<0.001	5625	2817	2808	0.855
Yes	6831	2991	3840		4225	2108	2117	

Abbreviations: Estimate, the number of enrollments; LN, lymph node; No, Patients without perioperative chemotherapy; NSCLC, non‐small cell lung cancer; PSM, propensity score matching; Sub, Sublobar lung resection; Yes, Patients with perioperative chemotherapy.

As shown in Table 1, after PSM, there were no significant differences in age, sex, race, histologic type, location of primary tumor, number of resected LNs, surgery type, stage, and LN metastases between the perioperative chemotherapy group and the surgery alone group.

### 3.2 Survival outcome

**FIGURE 1:**
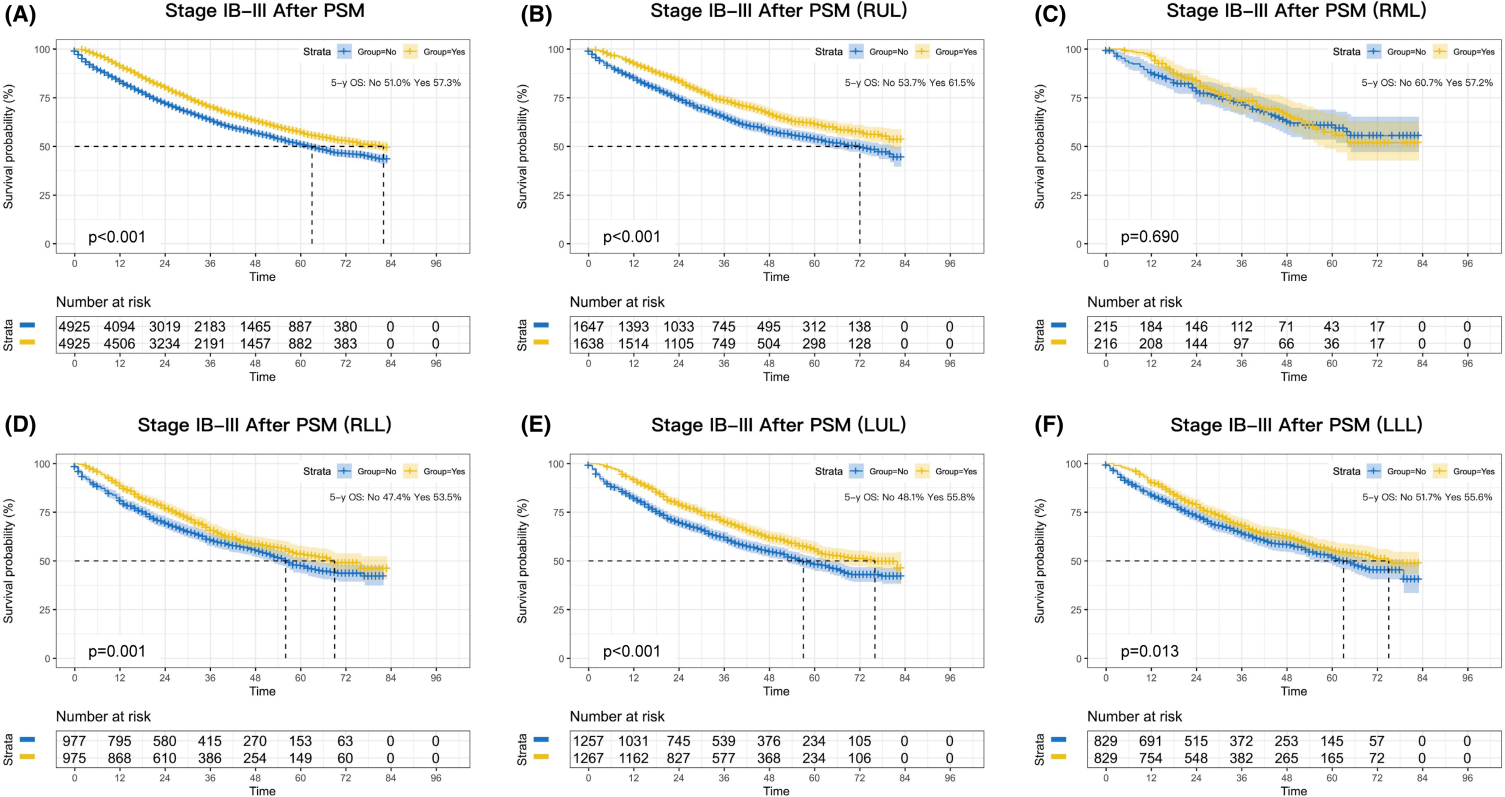
Kaplan–Meier survival curves after perioperative chemotherapy or surgery alone in NSCLC patients with stage IB‐III after PSM (A). Survival curves for RUL (B), RML (C), RLL (D), LUL (E), and LLL (F) after PSM.

Patients who received perioperative chemotherapy had a significant better OS than those who received surgery alone (*p* < 0.001; Figure S2A). The absolute improvement in 5‐year survival was 1.9%. After PSM, the results indicated that perioperative chemotherapy could significantly benefit patients with IB–III disease (*p* < 0.001; Figure 1A), and the absolute improvement in 5‐year survival was 6.3%, which greater than the result before PSM.

To further explore the benefit of perioperative chemotherapy, lobar subgroup analyses were performed. As shown in Figure 1B–F, perioperative chemotherapy was associated with a significantly better OS than surgery alone in most lobar subgroups except the RML subgroup (*p* = 0.690). The absolute improvement was 7.8% for RUL (*p* < 0.001), 6.1% for RLL (*p* = 0.001), 7.7% for LUL (*p* < 0.001), and 3.9% for LLL (*p* = 0.013).

### 3.3 Survival outcome according to stage

**FIGURE 2:**
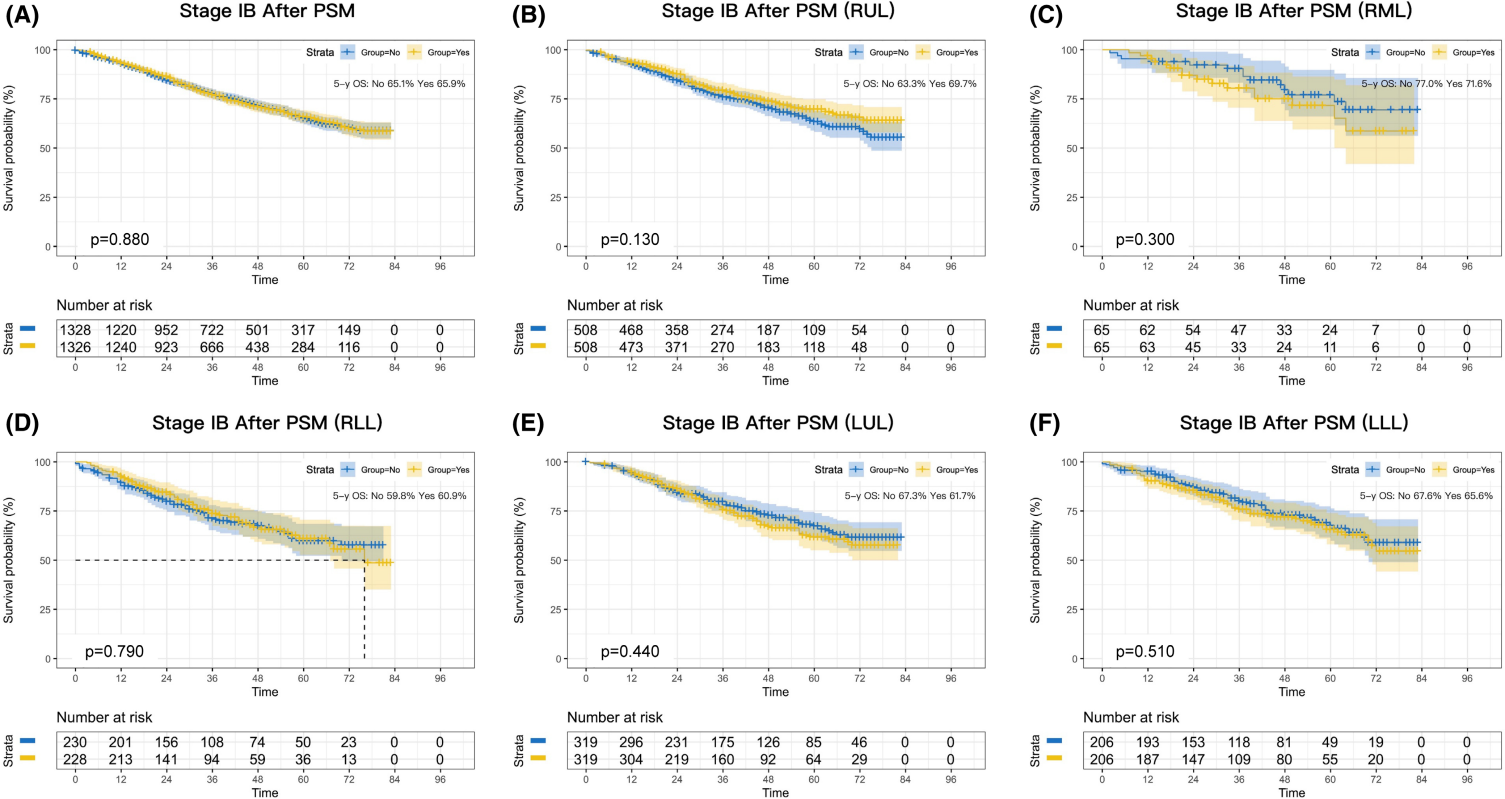
Kaplan–Meier survival curves after perioperative chemotherapy or surgery alone in NSCLC patients with stage IB after PSM (A). Survival curves for RUL (B), RML (C), RLL (D), LUL (E), and LLL (F) after PSM.

Next, we performed survival analysis according to stage subclassification (stage IB, stage II, or stage III). A total of 11,174 patients with stage IB were enrolled in the study. Before PSM, there was an absolute improvement in 5‐year survival of 6% after perioperative chemotherapy compared with surgery alone (*p* = 0.001; Figure S2B). However, the benefit disappeared after PSM (*p* = 0.880; Figure 2A). Lobar subgroup analyses did not identify a potential benefit of a lobar subgroup (Figure 2B–F).

**FIGURE 3:**
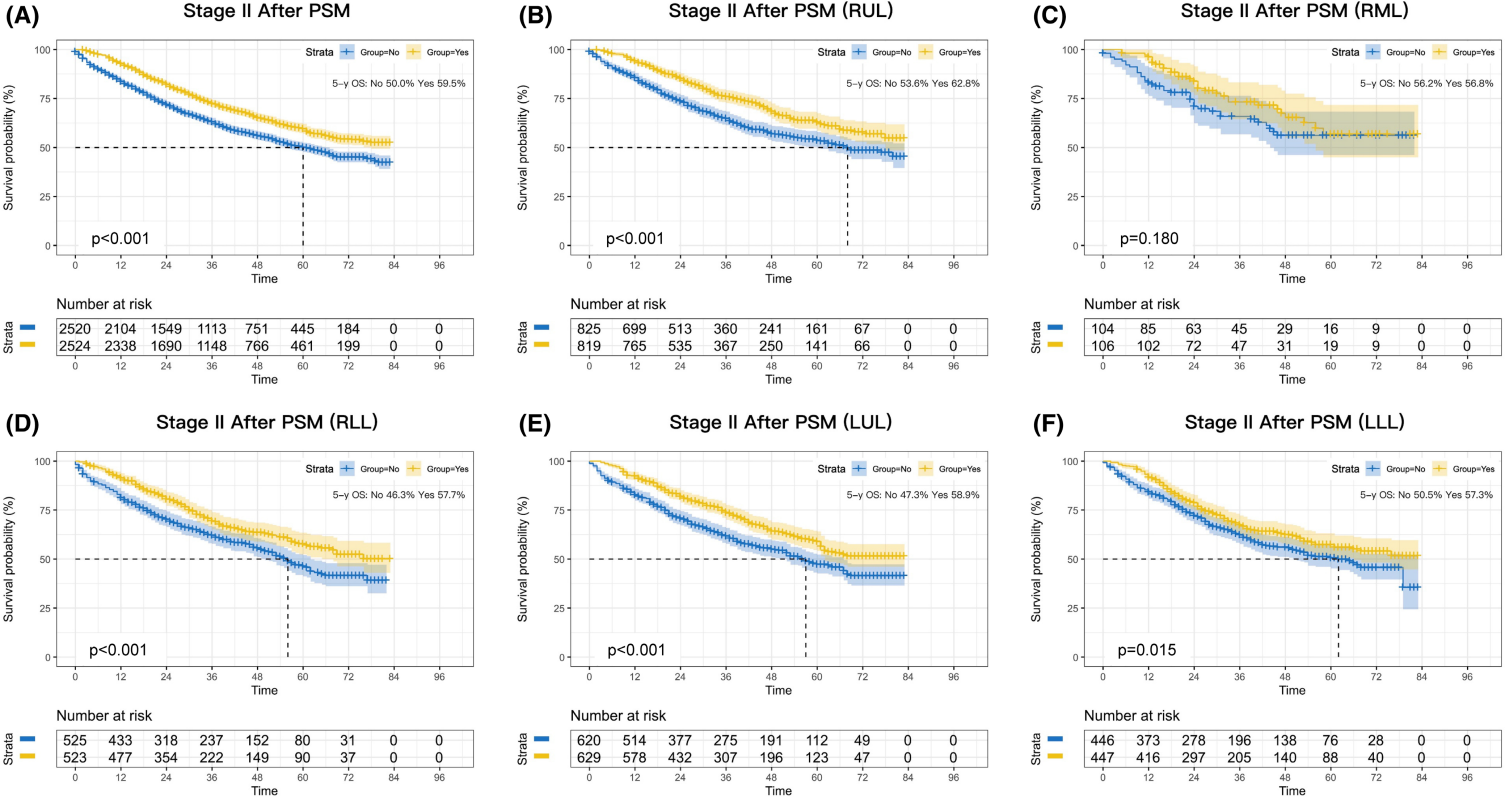
Kaplan–Meier survival curves after perioperative chemotherapy or surgery alone in NSCLC patients with stage II after PSM (A). Survival curves for RUL (B), RML (C), RLL (D), LUL (E), and LLL (F) after PSM.

Consistently significant survival benefits were observed in stage II patients before PSM (*p* < 0.001; Figure S2C) and after PSM (*p* < 0.001; Figure 3A), and the absolute improvement in 5‐year survival was 10.5% and 9.5%, respectively. Lobar subgroup analyses demonstrated that perioperative chemotherapy significantly benefited patients with RUL, RLL, LUL, and LLL tumors (*p* < 0.001, *p* < 0.001, *p* < 0.001, and *p* = 0.015, respectively; Figure 3B,D–F), whereas no benefit was observed for patients in the RML subgroup (*p* = 0.180; Figure 3C).

**FIGURE 4:**
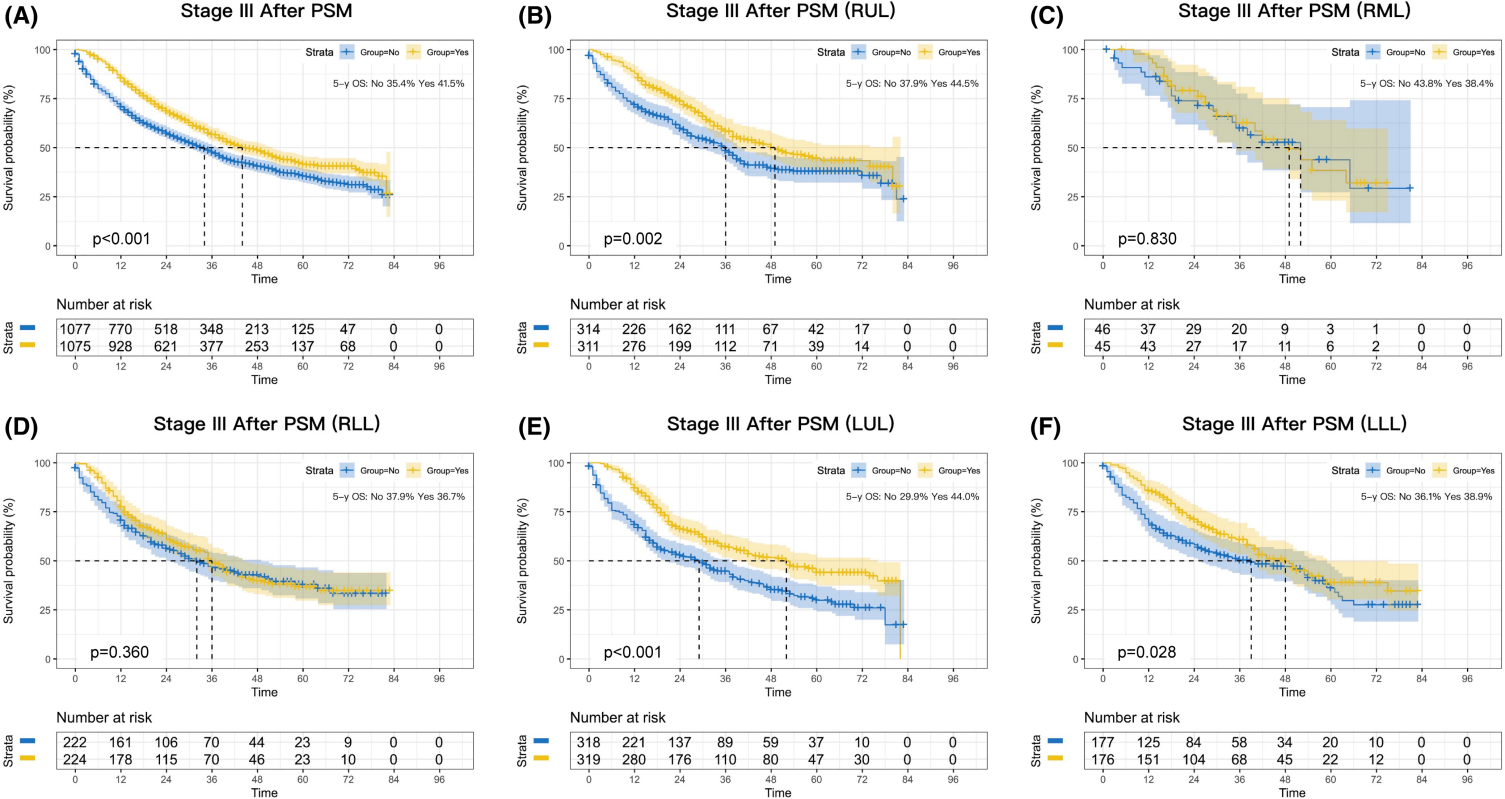
Kaplan–Meier survival curves after perioperative chemotherapy or surgery alone in NSCLC patients with stage III after PSM (A). Survival curves for RUL (B), RML (C), RLL (D), LUL (E), and LLL (F) after PSM.

A statistically significant OS benefit was observed for perioperative chemotherapy in stage III patients, with an absolute improvement in 5‐year OS of 5.8% before PSM (*p* < 0.001; Figure S2D) and 6.1% after PSM (*p* < 0.001; Figure 4A). Stage III patients in the RML and RLL subgroups did not significantly benefit from perioperative chemotherapy (*p* = 0.830 and *p* = 0.360, respectively; Figure 4C,D), whereas patients in the RUL, LUL, and LLL subgroups did (*p* = 0.002, *p* < 0.001, and *p* = 0.028; Figure 4B,E–F).

### 3.4 Survival according to age after PSM

As shown in Figure S3, perioperative chemotherapy resulted in a better OS in both the younger and older groups (*p* < 0.001 both), although the survival benefit was smaller in younger people than in older people.

### 3.5 Survival according to histologic type after PSM

Survival analyses according to histological type showed that LUSC and LUAD patients who received perioperative chemotherapy had a better OS (*p* < 0.001 both; Figure S4A,B), whereas patients with OC (including large cell carcinoma, carcinoid, and other NSCLCs) did not benefit from perioperative chemotherapy (*p* = 0.460; Figure S4C).

### 3.6 Survival according to LN metastases

As shown in Figure S5, patients with LN metastases had a significant greater survival benefit after perioperative chemotherapy than patients without LN metastases, with an absolute improvement of 11% (*p* < 0.001) and 3% (*p* = 0.015).

### 3.7 Survival according to sex

The results of analysis of survival according to sex are shown in Figure S6. Men had better OS after perioperative chemotherapy than nonperioperative chemotherapy (*p* < 0.001), and a similar positive effect was observed in women (*p* < 0.001).

### 3.8 Cox regression analysis

**FIGURE 5:**
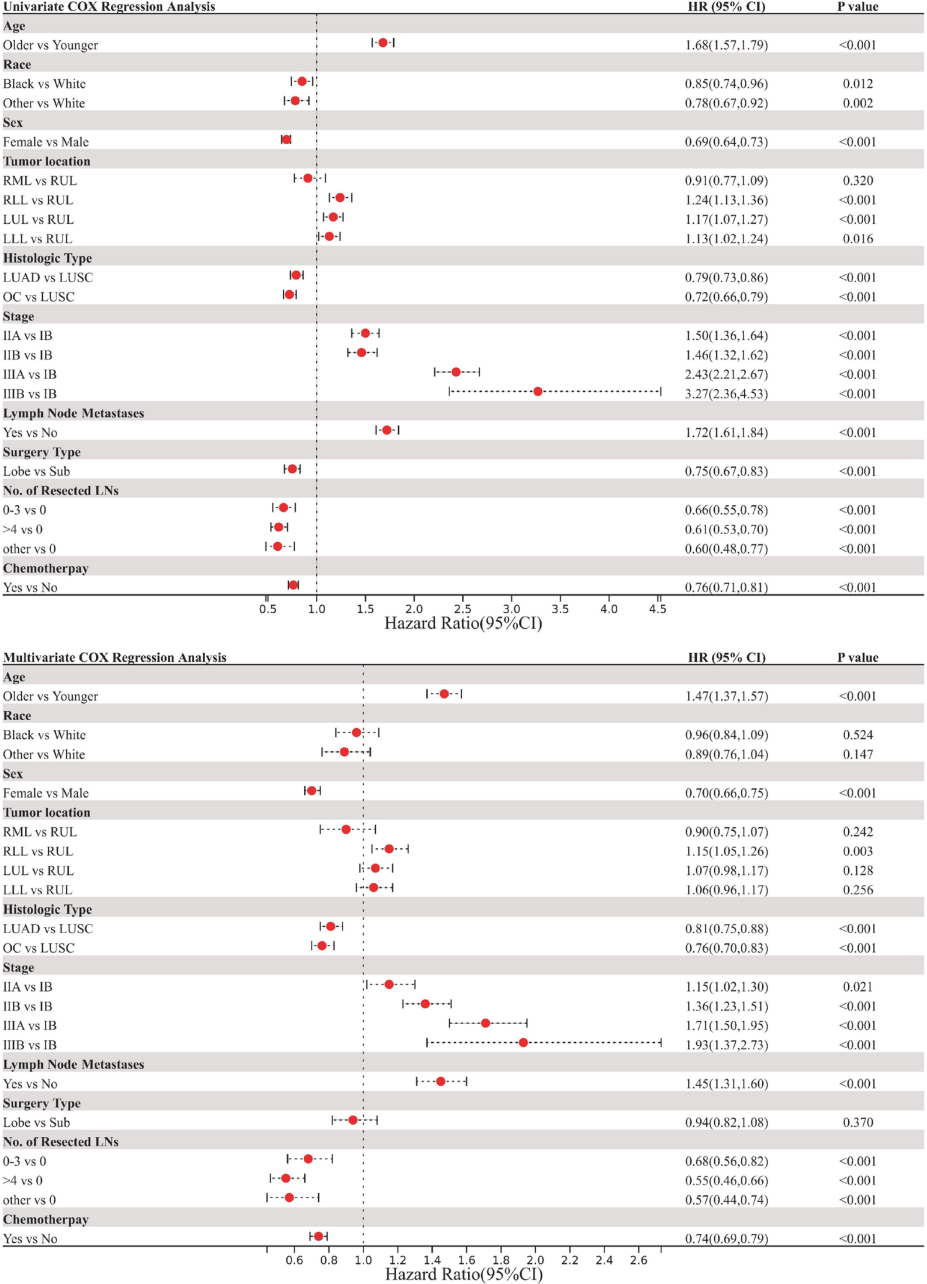
Cox regression analyses of factors affecting overall survival in patients with stage IB‐III.

To explore any potentially confounding factors related to OS, Cox regression analysis was performed. The HRs, p values, and 95% CIs are summarized in Table S1. Univariate analysis demonstrated that older age, white people, male sex, LUSC, advanced stage, LN metastases, sublobar resection, no LN dissection, and no perioperative chemotherapy were associated with worse OS (Figure 5). Similarly, multivariate Cox regression analysis revealed that older age, male sex, LUSC, advanced stage, LN metastases, no LN dissection, and no perioperative chemotherapy were associated with poor OS (Figure 5).

## 4 DISCUSSION

Over the past two decades, perioperative chemotherapy has become a standard treatment for NSCLC, and it has benefited many early‐stage patients with resectable tumors.^18^ However, whether adjuvant chemotherapy, a type of perioperative chemotherapy, benefits stage IB patients remains controversial because of confounding results. Gary's study, a mature analysis of Cancer and Leukemia Group B (CALGB) 9633, is the only RCT designed specifically for stage IB NSCLC patients. It showed no difference in survival between the adjuvant chemotherapy group and the nonadjuvant chemotherapy group (HR, 0.83; CI, 0.64–1.08; *p* = 0.120).^19^ However, exploratory subgroup analysis in the CALGB 9633 trail showed that patients with stage IB with tumors >4 cm in diameter had a significantly better OS (HR, 0.69; CI, 0.48–0.99; *p* = 0.043). Several RCTs including IALT,^7^ NCIC‐CTG‐JBR‐10,^9^ and ANITA^8^ showed a significant OS advantage of adjuvant chemotherapy in stage II‐IIIA patients, but no significant OS advantage in the stage IB subset. The study by Harubumi et al. was the only RCT that showed a statistically significant benefit of adjuvant chemotherapy in stage IB patients. However, the patients received uracil–tegafur, which is not approved in the USA and Europe. To date, no cisplatin‐based RCTs have demonstrated a significant OS advantage of adjuvant chemotherapy in the stage IB subset.

Unlike adjuvant chemotherapy, neoadjuvant chemotherapy, the other part of perioperative chemotherapy, has not been evaluated extensively. There are no studies assessing the effect of neoadjuvant chemotherapy specifically in stage IB patients. In Scagliotti's study,^20^ stage IB/IIA group (89.8% for IB and 10.2% for IIA), OS (HR, 1.02; 95% CI, 0.58–1.19; log‐rank *p* = 0.940) and PFS (HR, 1.06; 95% CI, 0.65–1.72; *p* = 0.830) were not significantly different between treatment groups (neoadjuvant chemotherapy vs. surgery alone), whereas a positive effect of neoadjuvant chemotherapy on OS and PFS was revealed in the IIB/IIIA group (HR, 0.42, 95% CI, 0.25–0.71, *p* < 0.001; HR, 0.51, 95% CI, 0.32–0.80, *p* = 0.002). In addition, a meta‐analysis^13^ of 15 RCTs (2385 patients with stage IB to IIIA) showed a significant benefit of neoadjuvant chemotherapy regarding survival (HR, 0.87, 95% CI 0.78–0.96, *p* = 0.007) and an absolute survival improvement at 5 years of 5% for all stages, which was similar to the survival benefit with adjuvant chemotherapy.^6^ Overall, perioperative chemotherapy, regardless of whether neoadjuvant chemotherapy or adjuvant chemotherapy, plays an important role in the systemic treatment of NSCLC and significantly benefits patients. However, there is no clear evidence of the positive effect of perioperative chemotherapy in patients with stage IB disease.

This retrospective study showed that patients who received perioperative chemotherapy had a better OS than those who underwent surgery alone including lobectomy and sublobar resection. However, the survival benefit was not significant in patients with IB disease. These results are consistent with those of previous studies. In this study, the absolute improvement in 5‐year OS after PSM was 6.3%, which was higher than the improvement reported in other studies, including the LACE Collaborative Group study (5.4% for adjuvant chemotherapy) and NSCLC Meta‐analysis Collaborative Group study (5% for neoadjuvant chemotherapy).^6^, ^13^ The difference between this study and previous reports may be related to differences in the patient population enrolled. Patients with stage I (IA/IB) accounted for 49.4% in the NSCLC Meta‐analysis Collaborative Group study and 37.5% in the LACE Collaborative Group study, whereas they accounted for 26.9% in this study after PSM. As reported previously, perioperative chemotherapy showed no meaningful clinical benefit in this population, suggesting that the large proportion of patients with stage I would influence the final absolute improvement in the LACE Collaborative Group study and the NSCLC Meta‐analysis Collaborative Group study. Furthermore, both patients who received neoadjuvant chemotherapy and adjuvant chemotherapy were included in this study because of the limitations of the SEER database, which may also have contributed to the discrepancy in the final result.

Primary tumor location may affect the prognosis of NSCLC after surgery, radiotherapy, and immunotherapy.^15^, ^16^, ^17^, ^21^, ^22^ Shaverdian et al.^16^ retrospectively analyzed 122 cases and showed that a lower lobe tumor location was associated with poor OS among all patients and those with biopsy‐confirmed disease. Similar results were reported by Stran et al. after Cox analysis^22^; patients whose primary tumor was located in the lower lobes had a worse OS than those with tumors in upper lobes after surgery (HR, 1.17; 95% CI, 1.03–1.32; *p* < 0.001). Thus, lobe‐specific analysis was performed in this study to explore whether tumor location affects the prognosis of NSCLC after perioperative chemotherapy and to identify the population that could benefit from perioperative chemotherapy. Previous studies classify RML into upper and lower groups to determine the effect on prognosis by Cox analysis. In this study, we consider RML as a subgroup to analyze the survival difference between perioperative chemotherapy and surgery alone, and found that patients with RML tumors did not significantly benefit from perioperative chemotherapy regardless of stage (stage IB–III), which is the first time this result is reported. The outcome of RML was reported to be the worst among all locations of NSCLC, which makes it unique and worthy of more attention.^23^, ^24^, ^25^ In addition, patients with stage III whose primary tumor located in RLL do not have a better OS after perioperative chemotherapy. These results challenge the hypothesis that the prognosis is similar for different lobes treated with the same strategy. However, the reasons that different lobes have different prognoses remain unknown. However, our previous study^26^ shed light on the different survival outcomes of NSCLC patients with different tumor location after sublobar lung resection, which may explain the reason of different outcomes in this study. Moreover, several studies found postoperative complications and postoperative respiratory failure predominantly develop in lung cancer patients with lower lobes,^27^, ^28^ which also contributed to the different survival outcomes among patients with different tumor location. In addition, study by Okamoto et al. revealed that tumor mutation burden (TMB) level differed according to tumor location^29^ in lung cancer. As a biomarker for the efficacy of chemotherapy in lung cancer,^30^ TMB level may differ in RUL, RML, RLL, LUL, and LLL. We speculate that this may also play a critical role in the different survival outcomes of NSCLC patients with different tumor location in our study.

To the best of our knowledge, this study is the first to discuss the difference between perioperative chemotherapy and surgery alone for NSCLC in different lung lobes. The study had several limitations. Firstly, although PSM was performed, there are still some unavoidable biases. Secondly, the SEER database provided no information on the regime on chemotherapy, such as dose and scheme. These factors may also influence the effect of perioperative chemotherapy. In addition, preoperative and postoperative CT would bring about different outcome in the surgery‐oriented treatments owing to their different mechanisms; however, we could not further analyze separately because the SEER database did not provide the information, which is also one of the limitations of our study. Meanwhile, the patient number of certain subgroup is low, especially for RML, which may lead to an inaccurate conclusion, therefore, results in RML needed to be validated in a larger sample size cohort. Lastly, due to inability to distinguish between neoadjuvant and adjuvant populations, we were unable to validate our findings in these two populations separately.

## 5 CONCLUSION

During the past decades, detecting technology and treatment in lung cancer have been developing rapidly with the rapid developments of precision medicine. For example, targeted therapy can help those with specific gene mutation and gain a better prognosis. Biomarker, such as TMB, can distinguish patient who may benefit from PD‐1/PD‐L1 inhibitor treatment. And our study, which is still needed to be verified in a larger cohort, may also guide physicians to provide a precise perioperative treatment strategy in early‐stage NSCLC. In conclusion, we proposed a precise lobe‐specific perioperative chemotherapy. For stage IB NSCLC, right middle lobe NSCLC from stage IB‐III and right lower lobe NSCLC from stage III, perioperative chemotherapy might not confer survival benefits.

## AUTHOR CONTRIBUTIONS

**Xi Lei:** Conceptualization (equal); data curation (equal); resources (equal); software (equal). **Tong Li:** Conceptualization (equal); data curation (equal); formal analysis (equal); funding acquisition (equal); investigation (equal); resources (equal); software (equal); supervision (equal). **Fuling Mao:** Conceptualization (equal); data curation (equal); resources (equal); software (equal). **Fan Ren:** Data curation (equal); formal analysis (equal); supervision (equal); writing – original draft (equal). **Quanying Tang:** Data curation (equal); methodology (equal); software (equal); supervision (equal); validation (equal). **Weibo Cao:** Resources (equal); software (equal); writing – original draft (equal); writing – review and editing (equal). **Lingling Zu:** Methodology (equal); project administration (equal); resources (equal); supervision (equal); validation (equal); visualization (equal). **Song XU:** Conceptualization (equal); data curation (equal); formal analysis (equal); funding acquisition (equal); investigation (equal); methodology (equal); project administration (equal); resources (equal).

## FUNDING INFORMATION

The present study was funded by the National Natural Science Foundation of China (82172776), Tianjin Key Medical Discipline (Specialty) Construction Project (TJYXZDXK‐061B), and Diversified Input Project of Tianjin National Natural Science Foundation (21JCYBJC01770).

## CONFLICT OF INTEREST STATEMENT

The authors declare that they have no conflicts of interest regarding the content or publication of this paper.

## ETHICS STATEMENT

SEER data are available to eligible individuals from the National Cancer Institute (NCI) upon completion of a data use agreement. We completed a data use agreement to access the data reported in this manuscript. A data use agreement per NCI/SEER policies was signed in order to obtain approval to access deidentified SEER public‐use data through SEER*Stat.

## Supporting information


Figure S1

Figure S2

Figure S3

Figure S4

Figure S5

Figure S6

Table S1

## Data Availability

All data relevant to the study are all available through SEER*Stat.
