# Supplementary material for: Lobe‐specific analysis of perioperative chemotherapy for non‐small cell lung cancer patients
Source: Cancer Med. 2023 Jul 5;12(16):16896–905. doi: 10.1002/cam4.6319 (PMC10501251; doi:10.1002/cam4.6319)
Supplement: Supplementary file 2 — Figure S2 [file CAM4-12-16896-s003.pdf]

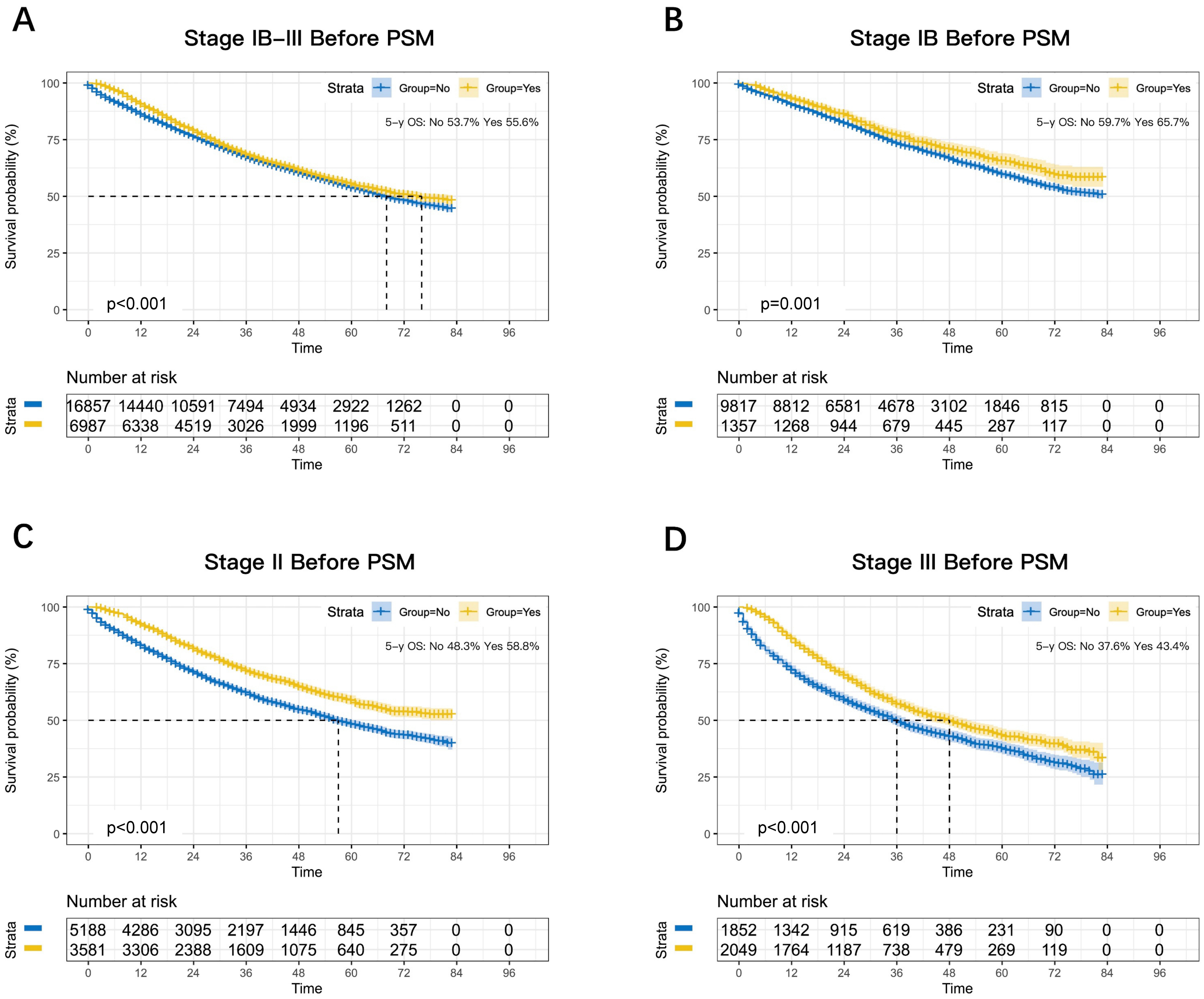
A
Stage IB-III Before PSM
Strata Group=No Group=Yes
5-y OS: No 53.7% Yes 55.6%
p<0.001
Survival probability (%)
Time
Number at risk
16857 14440 10591 7494 4934 2922 1262 0 0
6987 6338 4519 3026 1999 1196 511 0 0
B
Stage IB Before PSM
Strata Group=No Group=Yes
5-y OS: No 59.7% Yes 65.7%
p=0.001
Survival probability (%)
Time
Number at risk
9817 8812 6581 4678 3102 1846 815 0 0
1357 1268 944 679 445 287 117 0 0
C
Stage II Before PSM
Strata Group=No Group=Yes
5-y OS: No 48.3% Yes 58.8%
p<0.001
Survival probability (%)
Time
Number at risk
5188 4286 3095 2197 1446 845 357 0 0
3581 3306 2388 1609 1075 640 275 0 0
D
Stage III Before PSM
Strata Group=No Group=Yes
5-y OS: No 37.6% Yes 43.4%
p<0.001
Survival probability (%)
Time
Number at risk
1852 1342 915 619 386 231 90 0 0
2049 1764 1187 738 479 269 119 0 0
