# Supplementary figures and images for: Lobe‐specific analysis of perioperative chemotherapy for non‐small cell lung cancer patients
Source: Cancer Med. 2023 Jul 5;12(16):16896–905. doi: 10.1002/cam4.6319 (PMC10501251; doi:10.1002/cam4.6319)

A

≤ 70y

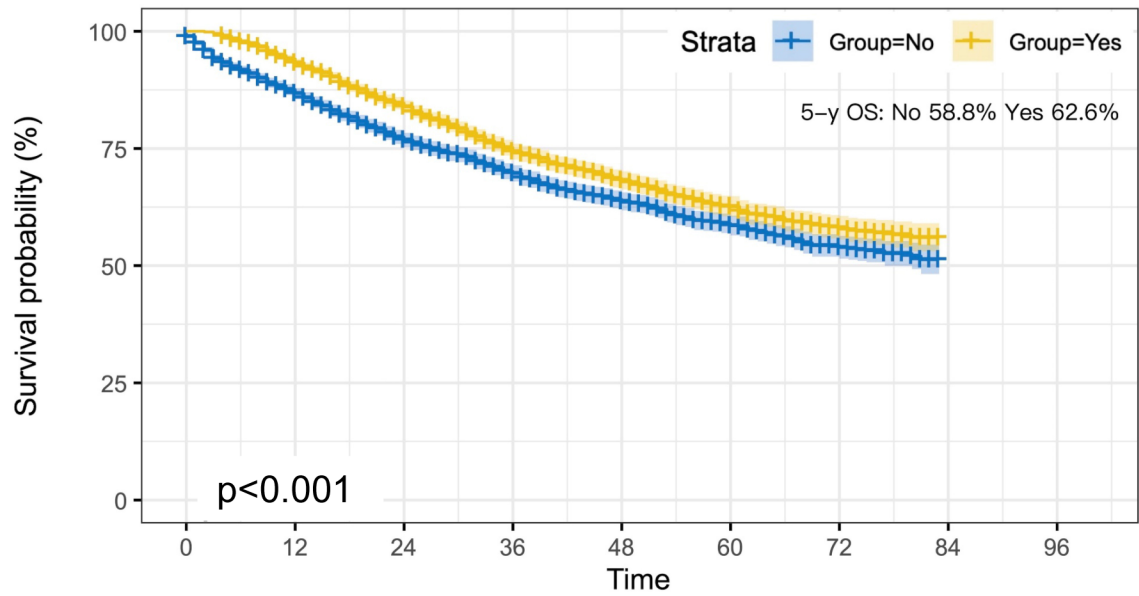

Number at risk

|        |  |      |      |      |      |      |     |     |    |    |
|--------|--|------|------|------|------|------|-----|-----|----|----|
| Strata |  | 0    | 12   | 24   | 36   | 48   | 60  | 72  | 84 | 96 |
|        |  | 3101 | 2666 | 2000 | 1509 | 1019 | 627 | 281 | 0  | 0  |
|        |  | 3089 | 2881 | 2132 | 1481 | 1008 | 625 | 279 | 0  | 0  |

Time

B

> 70y

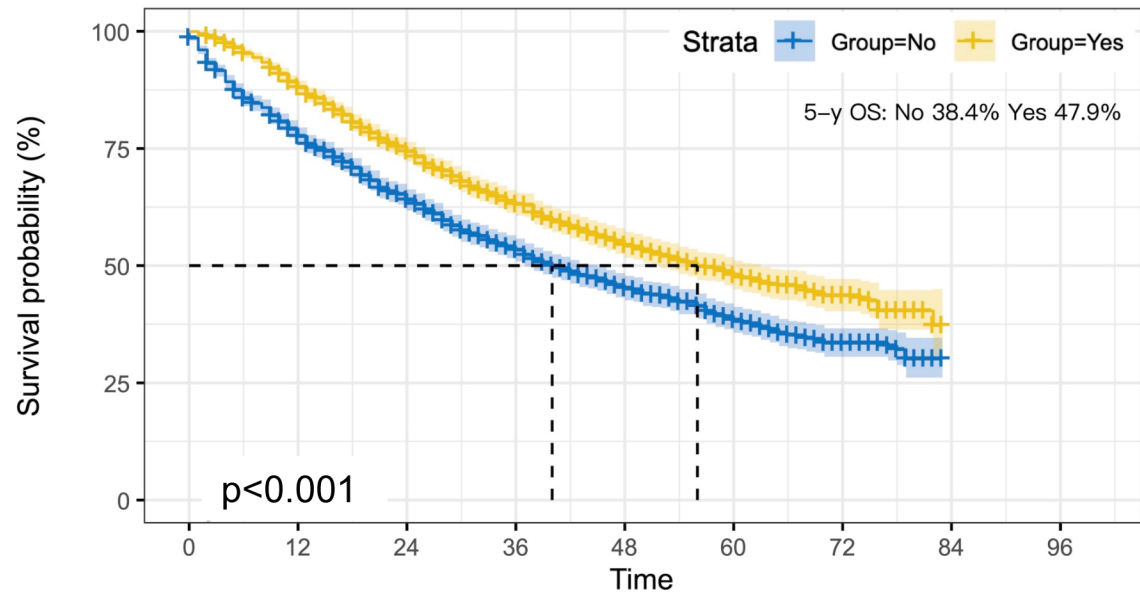

Number at risk

|        |  |      |      |      |     |     |     |     |    |    |
|--------|--|------|------|------|-----|-----|-----|-----|----|----|
| Strata |  | 0    | 12   | 24   | 36  | 48  | 60  | 72  | 84 | 96 |
|        |  | 1824 | 1428 | 1019 | 674 | 446 | 260 | 99  | 0  | 0  |
|        |  | 1836 | 1625 | 1102 | 710 | 449 | 257 | 104 | 0  | 0  |

Time

Supplement: Supplementary file 3 — Figure S3 [file CAM4-12-16896-s006.pdf]

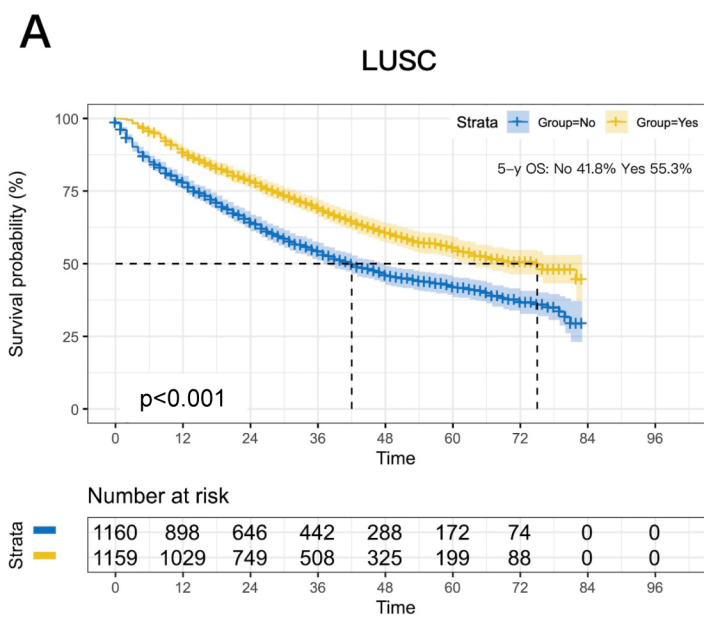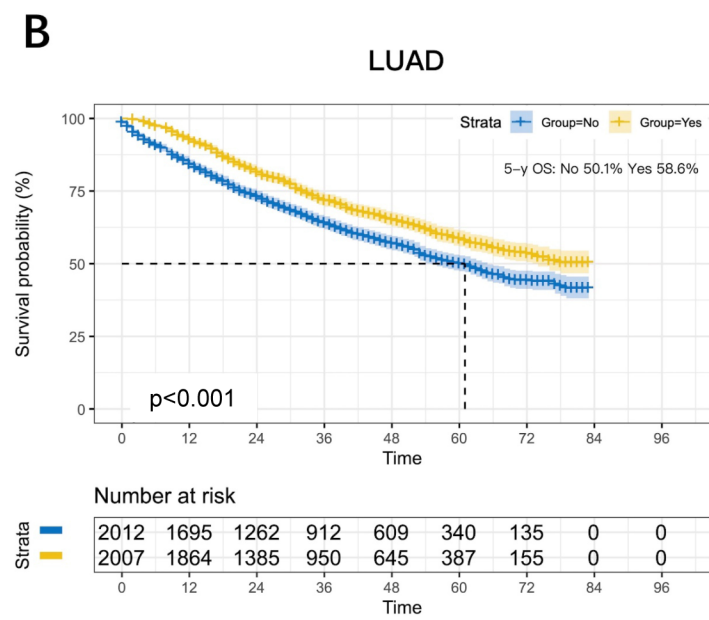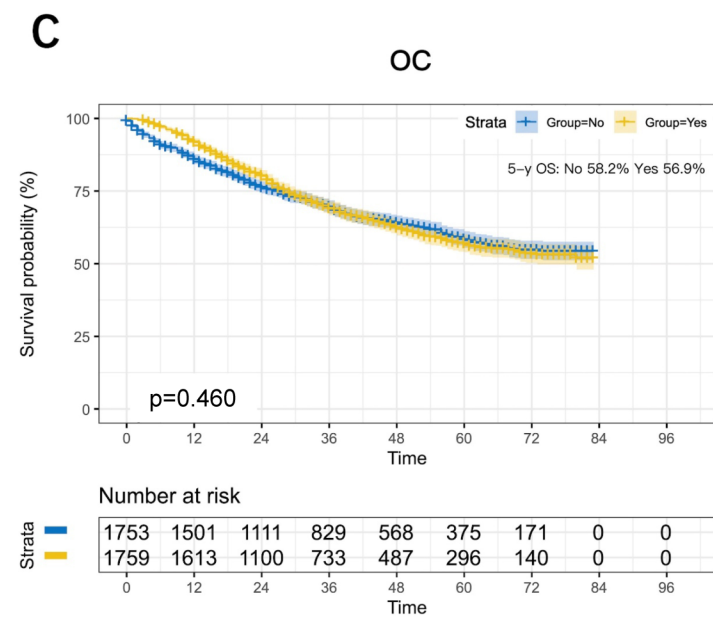

Supplement: Supplementary file 4 — Figure S4 [file CAM4-12-16896-s007.pdf]

A

no-LN Metastases

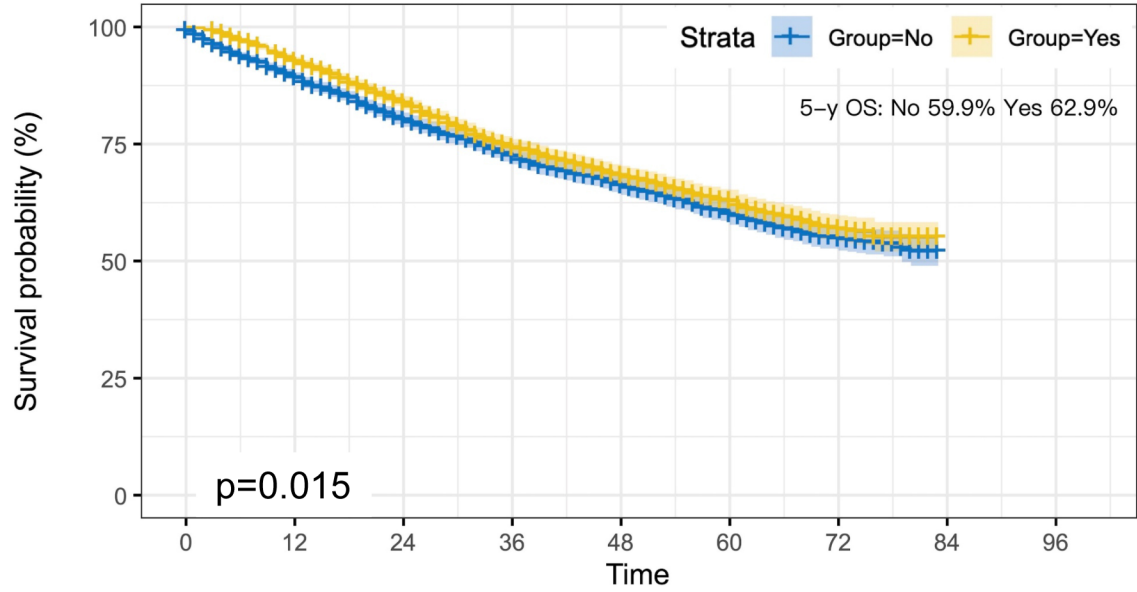

Number at risk

| Strata | Time |      |      |      |     |     |     |    |    |  |
|--------|------|------|------|------|-----|-----|-----|----|----|--|
|        | 0    | 12   | 24   | 36   | 48  | 60  | 72  | 84 | 96 |  |
| ■      | 2817 | 2493 | 1912 | 1419 | 985 | 595 | 269 | 0  | 0  |  |
| ■      | 2808 | 2602 | 1904 | 1318 | 878 | 555 | 226 | 0  | 0  |  |

B

LN Metastases

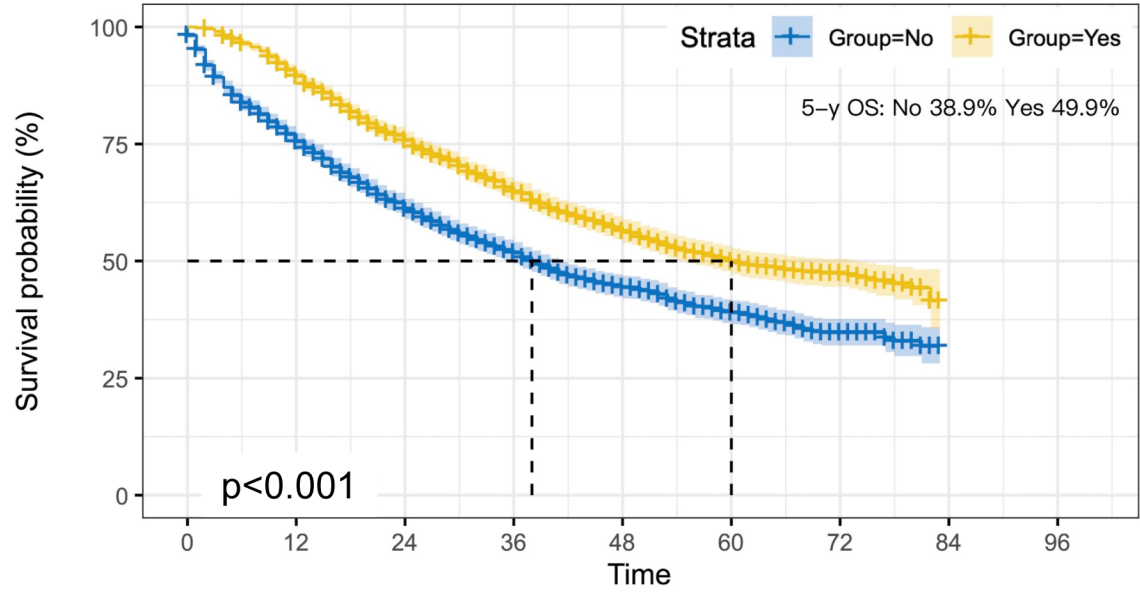

Number at risk

| Strata | Time |      |      |     |     |     |     |    |    |  |
|--------|------|------|------|-----|-----|-----|-----|----|----|--|
|        | 0    | 12   | 24   | 36  | 48  | 60  | 72  | 84 | 96 |  |
| ■      | 2108 | 1601 | 1107 | 764 | 480 | 292 | 111 | 0  | 0  |  |
| ■      | 2117 | 1904 | 1330 | 873 | 579 | 327 | 157 | 0  | 0  |  |

Supplement: Supplementary file 5 — Figure S5 [file CAM4-12-16896-s005.pdf]

A

Male

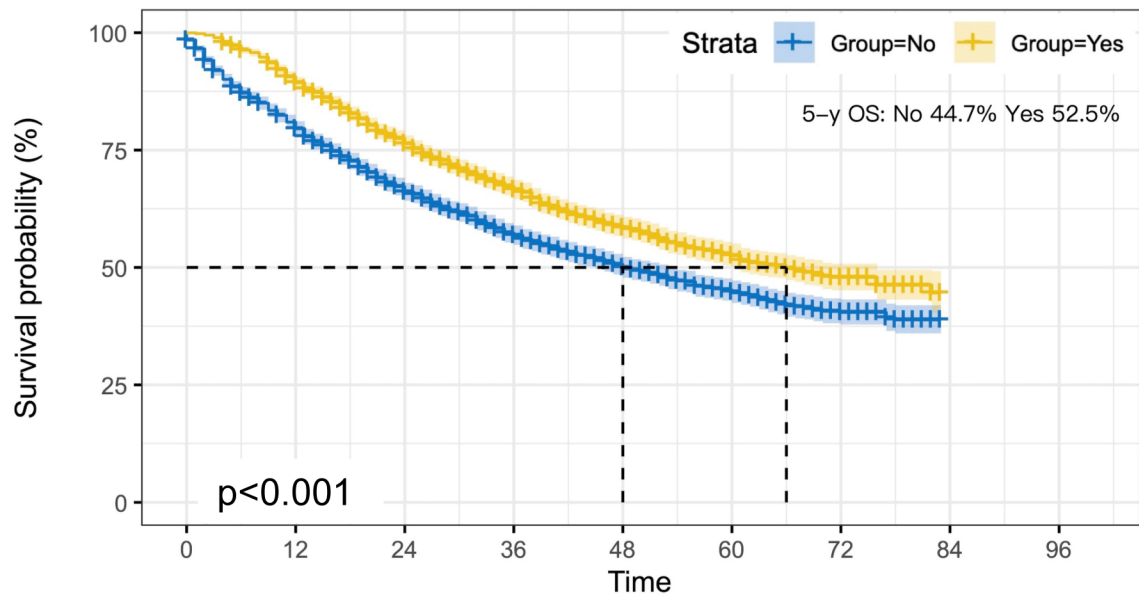

Number at risk

|        |      |      |      |      |      |     |     |     |    |    |
|--------|------|------|------|------|------|-----|-----|-----|----|----|
| Strata |      | 2484 | 1985 | 1416 | 989  | 655 | 371 | 171 | 0  | 0  |
|        |      | 2493 | 2240 | 1572 | 1046 | 687 | 421 | 186 | 0  | 0  |
|        | Time | 0    | 12   | 24   | 36   | 48  | 60  | 72  | 84 | 96 |

B

Female

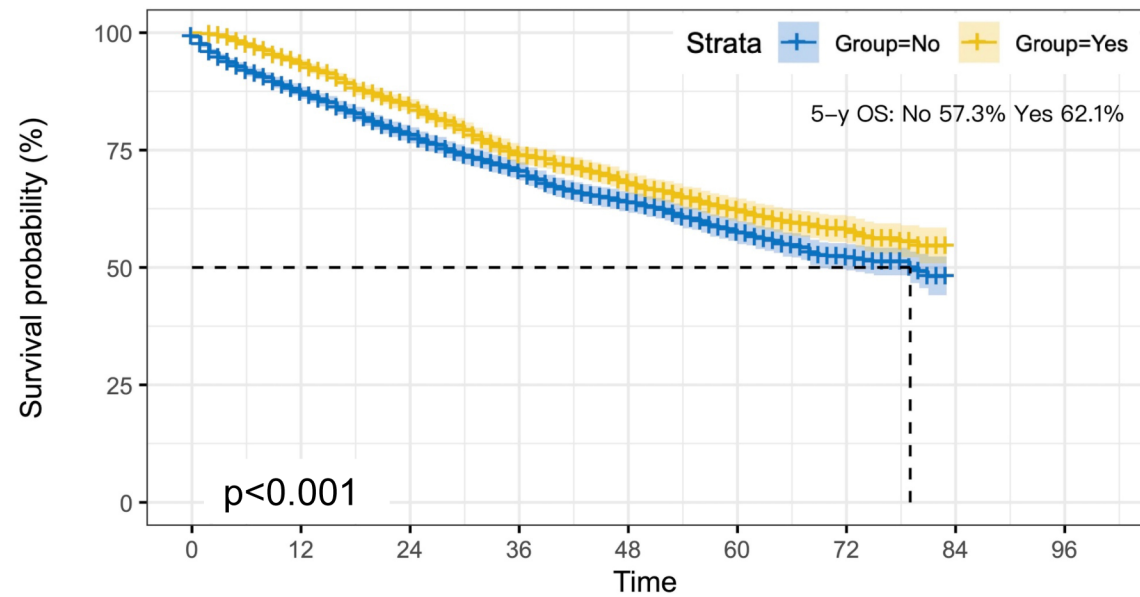

Number at risk

|        |      |      |      |      |      |     |     |     |    |    |
|--------|------|------|------|------|------|-----|-----|-----|----|----|
| Strata |      | 2441 | 2109 | 1603 | 1194 | 810 | 516 | 209 | 0  | 0  |
|        |      | 2432 | 2266 | 1662 | 1145 | 770 | 461 | 197 | 0  | 0  |
|        | Time | 0    | 12   | 24   | 36   | 48  | 60  | 72  | 84 | 96 |

Supplement: Supplementary file 6 — Figure S6 [file CAM4-12-16896-s004.pdf]
