# Supplementary material for: Lobe‐specific analysis of perioperative chemotherapy for non‐small cell lung cancer patients
Source: Cancer Med. 2023 Jul 5;12(16):16896–905. doi: 10.1002/cam4.6319 (PMC10501251; doi:10.1002/cam4.6319)
Supplement: Supplementary file 7 — Table S1 [file CAM4-12-16896-s002.docx]

| **Supplemental Table 1. Details of COX Regression Analysis** | | | | | |
| --- | --- | --- | --- | --- | --- |
| **Univariate COX Regression Analysis** | **HR** | **LOWER** | **UPPER** | **HR (95% CI)** | **P value** |
| **Age** |  |  |  |  |  |
| Older vs Younger | 1.68 | 1.57 | 1.79 | 1.68(1.57,1.79) | <0.001 |
| **Race** |  |  |  |  |  |
| Black vs White | 0.85 | 0.74 | 0.96 | 0.85(0.74,0.96) | 0.012 |
| Other vs White | 0.78 | 0.67 | 0.92 | 0.78(0.67,0.92) | 0.002 |
| **Sex** |  |  |  |  |  |
| Female vs Male | 0.69 | 0.64 | 0.73 | 0.69(0.64,0.73) | <0.001 |
| **Tumor location** |  |  |  |  |  |
| RML vs RUL | 0.91 | 0.77 | 1.09 | 0.91(0.77,1.09) | 0.320 |
| RLL vs RUL | 1.24 | 1.13 | 1.36 | 1.24(1.13,1.36) | <0.001 |
| LUL vs RUL | 1.17 | 1.07 | 1.27 | 1.17(1.07,1.27) | <0.001 |
| LLL vs RUL | 1.13 | 1.02 | 1.24 | 1.13(1.02,1.24) | 0.016 |
| **Histologic Type** |  |  |  |  |  |
| LUAD vs LUSC | 0.79 | 0.73 | 0.86 | 0.79(0.73,0.86) | <0.001 |
| OC vs LUSC | 0.72 | 0.66 | 0.79 | 0.72(0.66,0.79) | <0.001 |
| **Stage** |  |  |  |  |  |
| ⅡA vs ⅠB | 1.50 | 1.36 | 1.64 | 1.50(1.36,1.64) | <0.001 |
| ⅡB vs ⅠB | 1.46 | 1.32 | 1.62 | 1.46(1.32,1.62) | <0.001 |
| ⅢA vs ⅠB | 2.43 | 2.21 | 2.67 | 2.43(2.21,2.67) | <0.001 |
| ⅢB vs ⅠB | 3.27 | 2.36 | 4.53 | 3.27(2.36,4.53) | <0.001 |
| **Lymph Node Metastases** |  |  |  |  |  |
| Yes vs No | 1.72 | 1.61 | 1.84 | 1.72(1.61,1.84) | <0.001 |
| **Surgery Type** |  |  |  |  |  |
| Lobe vs Sub | 0.75 | 0.67 | 0.83 | 0.75(0.67,0.83) | <0.001 |
| **No. of Resected LNs** |  |  |  |  |  |
| 0-3 vs 0 | 0.66 | 0.55 | 0.78 | 0.66(0.55,0.78) | <0.001 |
| >4 vs 0 | 0.61 | 0.53 | 0.70 | 0.61(0.53,0.70) | <0.001 |
| other vs 0 | 0.60 | 0.48 | 0.77 | 0.60(0.48,0.77) | <0.001 |
| **Chemotherpay** |  |  |  |  |  |
| Yes vs No | 0.76 | 0.71 | 0.81 | 0.76(0.71,0.81) | <0.001 |
|  |  |  |  |  |  |
| **Multivariate COX Regression Analysis** | **HR** | **LOWER** | **UPPER** | **HR (95% CI)** | **P value** |
| **Age** |  |  |  |  |  |
| Older vs Younger | 1.47 | 1.37 | 1.57 | 1.47(1.37,1.57) | <0.001 |
| **Race** |  |  |  |  |  |
| Black vs White | 0.96 | 0.84 | 1.09 | 0.96(0.84,1.09) | 0.524 |
| Other vs White | 0.89 | 0.76 | 1.04 | 0.89(0.76,1.04) | 0.147 |
| **Sex** |  |  |  |  |  |
| Female vs Male | 0.70 | 0.66 | 0.75 | 0.70(0.66,0.75) | <0.001 |
| **Tumor location** |  |  |  |  |  |
| RML vs RUL | 0.90 | 0.75 | 1.07 | 0.90(0.75,1.07) | 0.242 |
| RLL vs RUL | 1.15 | 1.05 | 1.26 | 1.15(1.05,1.26) | 0.003 |
| LUL vs RUL | 1.07 | 0.98 | 1.17 | 1.07(0.98,1.17) | 0.128 |
| LLL vs RUL | 1.06 | 0.96 | 1.17 | 1.06(0.96,1.17) | 0.256 |
| **Histologic Type** |  |  |  |  |  |
| LUAD vs LUSC | 0.81 | 0.75 | 0.88 | 0.81(0.75,0.88) | <0.001 |
| OC vs LUSC | 0.76 | 0.70 | 0.83 | 0.76(0.70,0.83) | <0.001 |
| **Stage** |  |  |  |  |  |
| ⅡA vs ⅠB | 1.15 | 1.02 | 1.30 | 1.15(1.02,1.30) | 0.021 |
| ⅡB vs ⅠB | 1.36 | 1.23 | 1.51 | 1.36(1.23,1.51) | <0.001 |
| ⅢA vs ⅠB | 1.71 | 1.50 | 1.95 | 1.71(1.50,1.95) | <0.001 |
| ⅢB vs ⅠB | 1.93 | 1.37 | 2.73 | 1.93(1.37,2.73) | <0.001 |
| **Lymph Node Metastases** |  |  |  |  |  |
| Yes vs No | 1.45 | 1.31 | 1.60 | 1.45(1.31,1.60) | <0.001 |
| **Surgery Type** |  |  |  |  |  |
| Lobe vs Sub | 0.94 | 0.82 | 1.08 | 0.94(0.82,1.08) | 0.370 |
| **No. of Resected LNs** |  |  |  |  |  |
| 0-3 vs 0 | 0.68 | 0.56 | 0.82 | 0.68(0.56,0.82) | <0.001 |
| >4 vs 0 | 0.55 | 0.46 | 0.66 | 0.55(0.46,0.66) | <0.001 |
| other vs 0 | 0.57 | 0.44 | 0.74 | 0.57(0.44,0.74) | <0.001 |
| **Chemotherpay** |  |  |  |  |  |
| Yes vs No | 0.74 | 0.69 | 0.79 | 0.74(0.69,0.79) | <0.001 |
