# Supplementary material for: Lobe‐specific analysis of perioperative chemotherapy for non‐small cell lung cancer patients
Source: Cancer Med. 2023 Jul 5;12(16):16896–905. doi: 10.1002/cam4.6319 (PMC10501251; doi:10.1002/cam4.6319)
Supplement: Supplementary file 1 — Figure S1 [file CAM4-12-16896-s001.pdf]

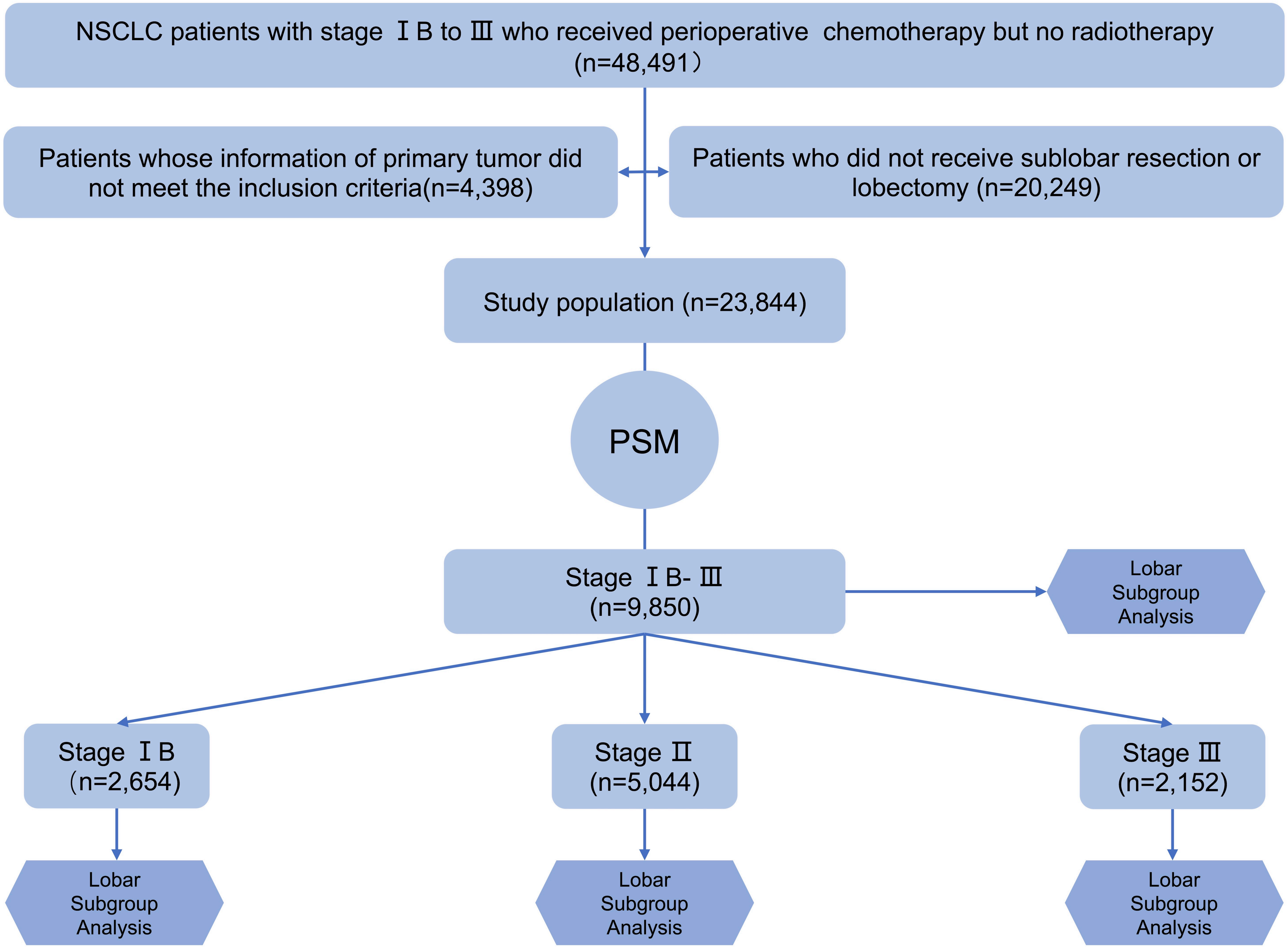
NSCLC patients with stage ⅠB to Ⅲ who received perioperative chemotherapy but no radiotherapy (n=48,491）
Patients whose information of primary tumor did not meet the inclusion criteria(n=4,398)
Patients who did not receive sublobar resection or lobectomy (n=20,249)
Study population (n=23,844)
PSM
Stage ⅠB- Ⅲ (n=9,850)
Lobar Subgroup Analysis
Stage ⅠB （n=2,654)
Stage Ⅱ (n=5,044)
Stage Ⅲ (n=2,152)
Lobar Subgroup Analysis
Lobar Subgroup Analysis
Lobar Subgroup Analysis
